# *Sedum* Growth Patterns under Different Pedoclimatic Conditions

**DOI:** 10.3390/plants12142739

**Published:** 2023-07-23

**Authors:** Alex-Péter Cotoz, Valentin-Sebastian Dan, Tincuța-Marta Gocan, Ileana Andreica, Sándor Rózsa, Maria Cantor

**Affiliations:** 1 Department of Horticulture and Landscape Design, University of Agricultural Sciences and Veterinary Medicine of Cluj-Napoca, 400372 Cluj-Napoca, Romania; alex.cotoz@usamvcluj.ro (A.-P.C.); dan.valentin@usamvcluj.ro (V.-S.D.); tincuta.gocan@usamvcluj.ro (T.-M.G.); rozsa.sandor@usamvcluj.ro (S.R.); 2 Department of Economics, University of Agricultural Sciences and Veterinary Medicine of Cluj-Napoca, 400372 Cluj-Napoca, Romania; iandreica@usamvcluj.ro

**Keywords:** succulents, abiotic stressors, development, growth media, propagation, roof gardens

## Abstract

This research paper presents a case study analysis of the behavior of three *Sedum* varieties and their growth in three different types of substrates without additional watering or fertilizing. The study aims to identify a suitable substrate for propagation and to provide insight into the plant’s growth patterns. By analyzing the growth of the *Sedum* species and varieties—SS’PW’, SS’CB’, and SS’P’—without intervening in their growth process, we were able to identify factors that play a more crucial role in promoting root growth, plant growth, aesthetic value, and use. Over a 20-month period, various technical tools were employed to conduct observations and measurements for both plants and weather conditions. The type of substrate significantly affected plant growth, with the green roof substrate exhibiting the highest overall average monthly root growth rate (0.92 ± 0.05 d, 1.01 ± 0.05 b, 0.96 ± 0.05 c) while in the case of stem growth, among all three varieties, the best results were obtained in the commercial mix (0.87 ± 0.04 a, 0.40 ± 0.02 c, 0.35 ± 0.02 d). Based on the morphological analyses, all values were significantly lower than the control. Best results for leaf weight and surface area were noticed in the green roof substrate with an average growth of 46%, 53%, 55%, and for stem weight, length, and thickness in the commercial mix with 64%, 61%, and 55% compared to the control, respectively. Leaves had varying morphological characteristics, but the chromatic characteristics were preserved. The plants had an overall poor growth which may not be desirable in landscape designs. The findings of this study are applicable in the planning and execution of eco-friendly infrastructure initiatives, leading to the development of more robust and environmentally friendly urban settings.

## 1. Introduction

Roof gardens have become an increasingly popular solution for addressing the negative effects of urbanization on the environment.

An extensive green roof is a lightweight system that typically consists of a thin layer of substrate (usually 5–15 cm deep) and drought-tolerant plants, such as sedums and grasses. This type of green roof requires minimal maintenance, is relatively inexpensive, and can be installed on a wide variety of buildings, including residential, commercial, and industrial structures. Extensive green roofs provide a number of benefits, such as reducing storm water runoff, improving air quality, and providing habitat for wildlife [1].

Succulent plants are a popular choice for green roofs because they are low maintenance, easy to use, and highly adaptable to various environmental conditions. Sedums in particular have gained popularity in recent years due to their remarkable adaptability to different environmental conditions. Their distinct metabolism and drought-tolerant nature make them ideal for use in locations where water availability may be limited [2].

To emphasize the hardiness of sedums throughout their growth cycle, we chose to utilize plant material in the early stages of development by propagating said plants. The most common method of propagating sedum plants is through vegetative means, either by division or by using stem or leaf cuttings. Plant propagation typically takes place during spring, specifically in May or late summer, around August, and can be carried out using whole leaves or flowerless shoot tips [3] and planted in a sandy substrate in order to help with the root growth [4].

These plants are particularly well-suited for growth in soils that lack nutrients [5]. Additionally, they prefer well-drained soils that allow excess water to flow away from their roots, reducing the risk of root rot.

For this study, sedums were used because of their drought resistance [6,7,8,9,10,11], adaptability to shallow substrates [12,13,14,15,16], persistence [17,18,19,20], and ability to reduce evapotranspiration during very hot periods [21,22,23,24]. For example, amongst the plant species examined under extreme hot and dry weather conditions, on a green roof*, Sedum pachyphyllum,* and *Sedum* × *rubrotinctum* proved to be the most successful in their growth. It was observed that these varieties also had the highest degree of succulence in their leaves even though no subsequent irrigation was made [25].

Several types of substrates have been used throughout the years in studies related to sedums. While some studies mentioned the use of naturally occurring growth media such as natural soil, organic matter, and other proprietary components, or crushed roof tiles in combination with generic substrates [13], others might have used a different approach by creating their special composition of 40% pumice, 40% thermally treated attapulgite clay, 8% peat, 7% compost, and 5% zeolite [15] or crushed brick, clay, pumice, and organic matter, and a recycled substrate composed of coarse pumice and municipal compost [26].

As a result of reviewing the scientific literature, there seems to be a consensus that most often, substrates used for research purposes reflect three typologies—one that mirrors a substrate as close as possible to the natural environment of the studied plant species; one rich in macro and microelements; and one deficient in nutrients. Thus, we could assume that the use of these typologies is preferred because they offer a diversified perspective and a wider spectrum of values, in response to the studied plants.

The current paper presents a case study analysis where the main objective was to assess the behavior of three sedum varieties (*Sedum spurium* ‘Purpur Winter’, *Sedum spathulifolium* ‘Cape Blanco’, and *Sedum spathulifolium* ‘Purpureum’) and their growth in three distinct types of substrates (green roof substrate, commercial mixture and river sand) without subsequent watering or fertilizing.

The research outcomes have practical implications for the planning and execution of eco-friendly infrastructure initiatives, particularly in regions characterized by hot and arid climates with water scarcity. The findings emphasize the need for careful plant selection considering not only theoretical attributes but also real-life examples and uses. The results of the study can help in the design and implementation of green infrastructure projects, helping to create more resilient and sustainable urban environments.

Future research should focus on assessing sedum performance under field conditions and exploring alternative approaches to enhance green roof sustainability and aesthetics. This could include investigating the effects of additional nutrients and irrigation strategies on sedum growth and exploring the use of different sedum varieties or other plant species with similar hardiness traits.

## 2. Results

### 2.1. Root Growth Rate

The data presented in Table 1, Table 2 and Table 3 illustrate the root growth patterns of *Sedum spurium* ‘Purpur Winter’ (S.S.’PW’), *Sedum spathulifolium* ‘Cape Blanco’ (S.S.’CB’), and *Sedum spathulifolium* ‘Purpureum’ (S.S.’P’) grown in three different substrates (green roof substrate, commercial mix, and river sand) for 20 months. The data include the total root length and monthly average root growth for each plant in each substrate.

#### 2.1.1. Green Roof Substrate (G.R.)

For *Sedum spurium* ‘Purpur Winter’ (SS’PW’), the first year saw a total root growth of 7.60 cm with a monthly average of 1.09 cm, while the second year had a root growth of 7.17 cm with a monthly average of 0.80 cm. The total root growth over 20 months was 14.77 cm.

For *Sedum spathulifolium* ‘Cape Blanco’ (SS’CB’), the first year resulted in a root growth of 8.26 cm with a monthly average of 1.18 cm, while the second year had a root growth of 7.96 cm with a monthly average of 0.88 cm. The total root growth over 20 months was 16.22 cm.

Finally, *Sedum spathulifolium* ‘Purpureum’ (SS’P’) had a root growth of 6.68 cm with a monthly average of 0.95 cm in the first year, and 8.63 cm with a monthly average of 0.96 cm in the second year. The monthly average difference was 0.21 cm, and the total root growth over 20 months was 15.31 cm.

#### 2.1.2. Commercial Mixture of Topsoil with Traces of Dolomite and Perlite (C.M.)

In the first year of study, SS ‘PW’ showed a total growth of 8.97 cm with a monthly average of 1.28 cm, while in the second year, it had a total growth of 7.71 cm with an average of 0.86 cm. The average monthly growth of SS ‘PW’ over the entire study period was 1.04 cm, and its root system showed a total growth of 16.68 cm.

Regarding SS’CB’, it exhibited a total growth of 6.92 cm with a monthly average of 0.99 cm in the first year, while in the second year, it had a total growth of 7.06 cm with an average of 0.78 cm. The average monthly growth of SS’CB’ over the entire study period was 0.87 cm, and its root system showed a total growth of 13.98 cm.

In the case of SS’P’, the first year of study showed a total growth of 5.11 cm with a monthly average of 0.73 cm, and in the second year, it had a total growth of 6.76 cm with an average of 0.75 cm. The average monthly growth of SS’P’ over the entire study period was 0.74 cm, and its root system showed a total growth of 11.87 cm.

#### 2.1.3. River Sand (R.S.)

*Sedum spurium* ‘Purpur Winter’ had a total root growth of 8.71 cm over a 20-month period with a higher monthly average root growth in the first year (0.88 cm).

*Sedum spathulifolium* ‘Cape Blanco’ had the highest total root growth of 14.21 cm. SS’CB’ had a higher monthly average root growth in the first year (1.01 cm) compared to the second year (0.79 cm).

*Sedum spathulifolium* ‘Purpureum’ had a total root growth of 12.20 cm. Additionally, this variety had a higher monthly average root growth in the first year (0.88 cm) compared to the second year (0.67 cm).

Among the experimental treatments, the monthly average growth of the radicular system was spread unevenly and was as follows: for SS’PW’ the highest value was recorded in C.M. with a monthly average of 1.04 cm, respectively, whereas in G.R. and R.S. the values were 11.54% and 48.08% smaller.

For SS’CB’ the highest value was noted in G.R., 1.01 cm, while in C.M. and R.S., the monthly average growth was smaller by a margin of 13.86% and 11.88%.

SS’P’ presented similar values to SS’CB’, where G.R. had the highest value of the three experimental treatments—0.96 cm monthly average. 22.92% and 20.83% higher than in C.M. and R.S., respectively.

**Figure 1:**
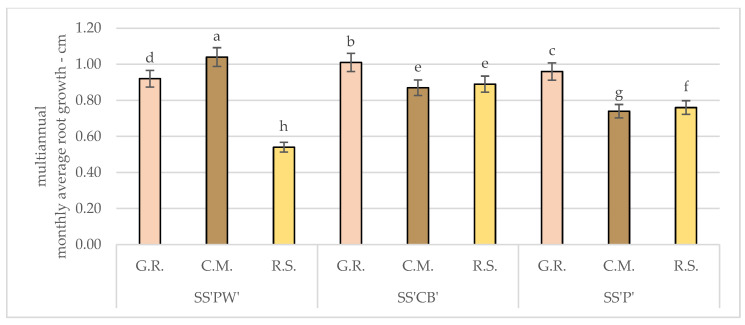
Average monthly root growth for S*edum* varieties under hydric and nutritive stress. Comparison between treatments. Bars represent the monthly average values. Different letters indicate significant differences between treatments (*p* < 0.05).

Considering the acquired experimental results from the three experimental treatments, represented in Figure 1, under the effect of nutritive and hydric stress factors, it appears that the plants grown in G.R. substrate had the highest total root growth, followed by the plants in C.M. and R.S. Additionally, it appeared that *Sedum spathulifolium* ‘Cape Blanco’ (SS’CB’) had the highest overall root growth.

Overall, the average root length in the green roof substrate was 8.10% higher than that in the commercial mixture and 24.11% higher than in the sand.

### 2.2. Stem Growth Rate

#### 2.2.1. Green Roof Substrate (G.R.)

The data presented in Table 4 pertain to the growth of three varieties of *Sedum* in green roof substrates specifically *Sedum spurium* ‘Purpur Winter’ (SS’PW’), *Sedum spathulifolium* ‘Cape Blanco’ (SS’CB’), and *Sedum spathulifolium* ‘Purpureum’ (SS’P’).

In the first year of the study, SS’PW’ grew a total of 4.30 cm with a monthly average of 0.61 cm, while in the second year, it grew a total of 1.52 cm with an average of 0.19 cm. The monthly average growth over the entire study period was 0.39 cm, and the monthly actual growth rate (A.G.R.) was on average 1.03 cm smaller than the expected growth rate (E.G.R). The stem length of SS’PW’ showed a total growth of 5.82 cm, which is 71% less than the expected growth.

Similarly, SS’CB’ grew a total of 1.65 cm with a monthly average of 0.24 cm in the first year, and in the second year, it grew a total of 1.27 cm with a monthly average of 0.16 cm. The monthly average growth over the entire study period was 0.19 cm, and A.G.R. showed a monthly average difference of 0.35 cm less than E.G.R. The stem length of SS’CB’ showed a total growth of 2.92 cm, which is 64% less than the expected growth.

Finally, SS’P’ grew a total of 1.25 cm with a monthly average of 0.18 cm in the first year, and in the second year, it grew a total of 1.21 cm with a monthly average of 0.15 cm. The average monthly growth over the entire study period was 0.16 cm, and A.G.R. showed a monthly average difference less than E.G.R. of 0.37 cm. The stem length of SS’P’ showed a total growth of 2.46 cm, which is 69% less than expected.

#### 2.2.2. Commercial Mixture of Topsoil with Traces of Dolomite and Perlite (C.M.)

Table 5 provides an analysis of the growth rate of the stem length of *Sedum* plants grown on a commercial mix culture substrate. *Sedum spurium* ‘Purpur Winter’ (SS’PW’) had a total growth of 6.10 cm in the first year with a monthly average of 0.87 cm and a total growth of 6.90 cm in the second year with an average of 0.86 cm. The monthly average growth rate over the entire study period was 0.87 cm. The relative growth rate difference (A.G.R.) was less than the relative decline rate (E.G.R.) by an average of 0.56 cm per month. However, the stem only grew a total of 13 cm, which is 35% less than the expected growth.

*Sedum spathulifolium* ‘Cape Blanco’ (SS’CB’) had a total growth of 2.63 cm in the first year with a monthly average of 0.38 cm and a total growth of 3.33 cm in the second year with an average of 0.42 cm. The average monthly growth rate over the entire study period was 0.40 cm. The A.G.R. was less than the E.G.R. by an average of 0.16 cm per month. The stem only grew a total of 5.96 cm, which is 25% less than the expected growth.

Finally, *Sedum spathulifolium* ‘Purpureum’ (SS’P’) had a total growth of 2.74 cm in the first year with a monthly average of 0.39 cm and a total growth of 2.50 cm in the second year with an average of 0.31 cm. The average monthly growth rate over the entire study period was 0.35 cm. The A.G.R. was less than the E.G.R. by an average of 0.18 cm per month. The stem grew a total of 5.24 cm, which is 34% less than the expected growth.

#### 2.2.3. River Sand (R.S.)

Based on the analysis of plant growth rates in R.S. substrate Table 6, we can make the following observations:

For *Sedum spurium* ‘Purpur Winter’ (SS’PW’), the total growth during the first year of the study was 4.64 cm with a monthly average of 0.66 cm. During the second year, the total growth was 2.70 cm with an average of 0.34 cm per month. The average monthly growth over the entire study period was 0.49 cm, and the monthly difference between the A.G.R. and E.G.R. was, on average, 0.93 cm. The stem length of the plant had a total growth of 7.34 cm, which was 63% less than expected.

For *Sedum spathulifolium* ‘Cape Blanco’ (SS’CB’), the total growth during the first year of the study was 1.52 cm with a monthly average of 0.22 cm. During the second year, the total growth was 1.41 cm with an average of 0.18 cm per month. The average monthly growth over the entire study period was 0.20 cm, and the monthly difference between the A.G.R. and E.G.R. was, on average, 0.35 cm. The stem length of the plant had a total growth of 2.93 cm, which was 63% less than expected.

For *Sedum spathulifolium* ‘Purpureum’ (SS’P’), the total growth during the first year of the study was 1.54 cm with a monthly average of 0.22 cm. During the second year, the total growth was 1.38 cm with an average of 0.17 cm per month. The average monthly growth over the entire study period was 0.19 cm, and the monthly difference between the A.G.R. and E.G.R. was, on average, 0.34 cm. The stem length of the plant had a total growth of 2.92 cm, which was 64% less.

**Figure 2:**
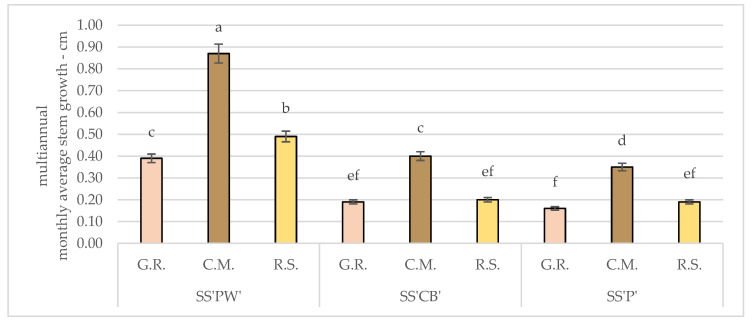
Monthly average stem growth for *Sedum* varieties under hydric and nutritive stress. Bars represent the monthly average values. Different letters indicate significant differences between treatments (*p* < 0.05).

According to Figure 2, among the experimental treatments, the monthly average growth of the radicular system was spread unevenly and was as follows: for SS’PW’ the highest value was recorded in C.M. with a monthly average of 0.87 cm, respectively, whereas in G.R. and R.S. the values were 55.17% and 43.68% smaller.

For SS’CB’ the highest value was noted in C.M., 0.40 cm, while in G.R. and R.S., the monthly average growth was smaller by a margin of 52.50% and 50%.

SS’P’ presented similar values to SS’CB’, where C.M. had the highest value of the three experimental treatments—0.35 cm monthly average. 54.29% and 45.71% higher than in G.R. and R.S., respectively.

We assumed that these results were affected by the existing nutrient deposits in the growth media, mainly due to the good potassium values, which are associated with the healthy functioning of plant metabolism, and hence photosynthesis.

### 2.3. Morphological Analysis

Table 7 presents the overall morphological characteristics of 1200 studied plant stems and how the studied plants developed compared to the control.

Considering that the plants were grouped into three experimental lots, in which the substrate factor varied, the lowest values of the studied plants were noted in the river sand (R.S.) lot with a growth percentage of 56% smaller than that of the control.

The second place was held by the green roof substrate (G.R.) with 50% of the control’s growth and in the first place was the commercial topsoil mix with traces of dolomite and perlite (C.M.) with 48%.

In terms of stem weight, there is a clear growth differentiation between the green roof plot—64% and the other two—59%.

On average, stem lengths were 67% shorter than those of control plants. The largest differences were found in the green roof substrate group, with an average of 73%.

In general, on average, the stem diameter values of all varieties in all experimental treatments were 12% smaller than those of the control—SS’PW’—11% of the total growth of the controls and SS’CB’ and SS’P’—12%.

Even though on average, compared to the control plants, the leaf areas studied were 65% smaller, the SS’PW’ specimens suffered the most, with an average growth of only 34% of the control plants. The most affected SS’PW’ specimens were those in the experimental sand group, with a growth of 15%. However, their weight was only 45% lower.

Although the different experimental plots showed different and varied results, the overall plant averages per species and plot are relatively close.

Concerning the previously reported data on plant morphology, for future reference, we further explore the detailed characteristics of total leaf and stem growth as these are relevant from an aesthetic point of view. The following data can provide valuable input on the aesthetic value of these plants grown in various pedo-climatic conditions.

**Figure 3:**
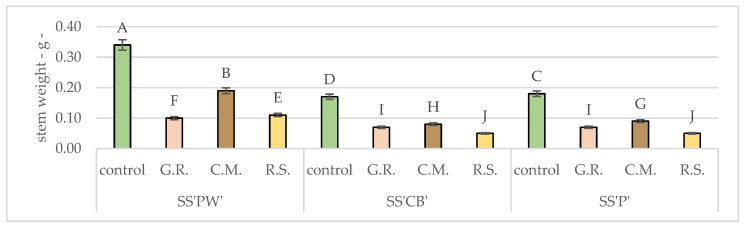
Average stem weight after 20 months of hydric and nutritive stress. Bars represent the average means expressed in grams. Different letters indicate significant differences between experimental treatments (*p* < 0.05).

**Figure 4:**
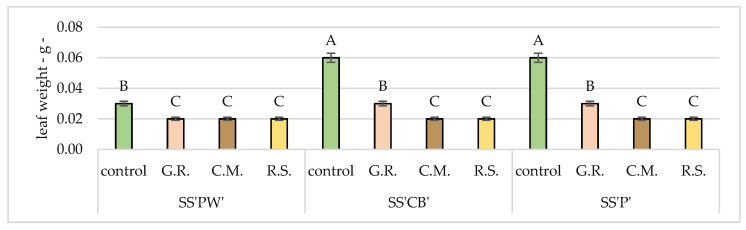
Average leaf weight after 20 months of hydric and nutritive stress. Bars represent the average means expressed in grams. Different letters indicate significant differences between experimental treatments (*p* < 0.05).

**Figure 5:**
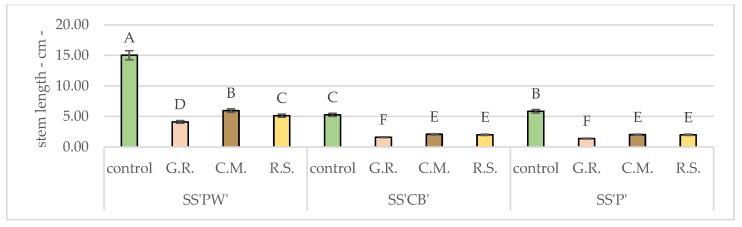
Average stem length after 20 months of hydric and nutritive stress. Bars represent the average means expressed in centimeters. Different letters indicate significant differences between experimental treatments (*p* < 0.05).

**Figure 6:**
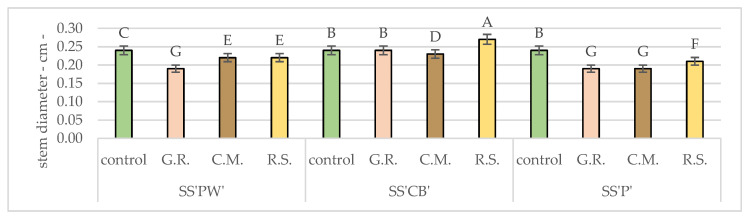
Average stem diameter after 20 months of hydric and nutritive stress. Bars represent the average means expressed in centimeters. Different letters indicate significant differences between experimental treatments (*p* < 0.05).

**Figure 7:**
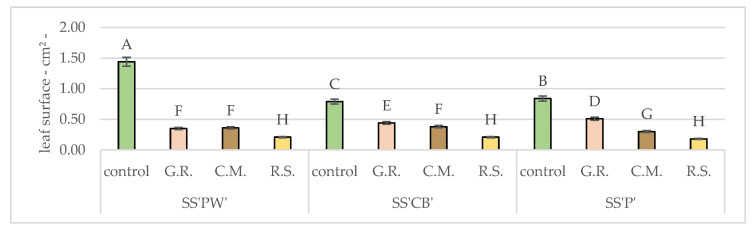
Average leaf surface after 20 months of hydric and nutritive stress. Bars represent the average means expressed in cm^2^. Different letters indicate significant differences between experimental treatments (*p* < 0.05).

The statistical analyses and results for the morphological measurements of the plants under study are depicted in Figure 3, Figure 4, Figure 5, Figure 6 and Figure 7. These figures offer detailed insights into various characteristics, namely stem weight, leaf weight, stem length, stem diameter and leaf surface area. By comparing the performance of each sedum variety across different substrates, these figures provide a comprehensive representation of their growth patterns with the control group.

The values presented in Figure 3, Figure 4, Figure 5, Figure 6 and Figure 7 and Table 8, Table 9, Table 10, Table 11 and Table 12 serve as informative indicators of the extent of growth decline associated with the use of specific substrates and lack of subsequent fertilization and irrigation. The findings reveal notable differences among plant varieties and substrate groupings. When considering overall growth, the control plants exhibited significantly superior growth compared to the experimental treatments consisting of the substrates G.R., C.M., and R.S and plant varieties SS’PW’, SS’CB’, and SS’P’.

The analysis depicted In Table 8 and Figure 3 examines the stem weight variations among our different sedum varieties and substrates. It provides insights into how specific substrates impact the growth of strong and sturdy stems in sedum plants. Among the varieties (SS’PW’, SS’CB’, SS’P’), the most favorable results, compared to the control, were observed in C.M (0.192 g, 0.083 g, 0.092 g), while R.S. exhibited decent results with only SS’PW’ (0.106 g). All values were significantly lower than the control.

Similarly, the leaf weight analysis presented in Table 9 and Figure 4 offers insights into how our substrates affected leaf growth, highlighting variations in fresh leaf weight. Among plant varieties (SS’PW’, SS’CB’, SS’P’), high values were observed in G.R. (0.021 g, 0.032 g, 0.032 g), contrasting with R.S. and C.M. where significant differences were observed—0.017 g, 0.024 g, 0.024 g and 0.017 g, 0.024 g, 0.025 g respectively. All values were significantly lower than the control.

The analytical results presented in Table 10 Figure 5 are dedicated to measuring the stems and how different substrates affected their elongation. Among varieties (SS’PW’, SS’CB’, SS’P’), the most favorable results, compared to the control, were observed in C.M (5.954 cm, 2.077 cm, 2.005 cm), while G.R. exhibited only mediocre results with the only highlight being SS’PW’ (4.100 cm). All values were significantly lower than the control.

Table 11 and Figure 6 present the analysis of stem diameter, which provides valuable information on the effect of substrates on the thickness and overall robustness of the sedum stems. By comparing the stem diameters of different sedum varieties, this analysis offers insights into the effect of substrates on stem growth. Opposed to previous results, in the case of stem diameter, the best results were noticed in R.S. for two out of the three plant varieties (SS’CB’—0.265 cm, SS’P’—0.211 cm) as opposed to the other substrates, which had significant variations. All other values were significantly lower than the control.

Lastly, the leaf surface area was examined in Table 12 and Figure 7, offering a comprehensive understanding of how the sedum varieties responded to various substrates in terms of leaf growth. The variations in leaf surface area demonstrate the effect of substrates on the expansion and growth of sedum leaves. For all three varieties (SS’PW’, SS’CB’, SS’P’), the best results compared to the control were obtained in G.R (0.345 cm^2^, 0.442 cm^2^, 0.505 cm^2^) as opposed to the lowest, R.S. (0.211 cm^2^, 0.209 cm^2^, 0.178 cm^2^). A small observation here has to be made related to SS’PW’, which had slightly better results in C.M. (0.361 cm^2^). All values were significantly lower than the control.

**Figure 8:**
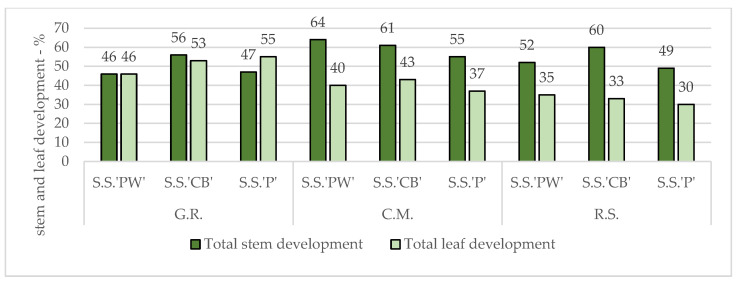
Overall morphological plant growth for *Sedum* plants under abiotic stress factors.

Figure 8 shows that all three plant varieties in G.R. exhibited similar values in both stem and leaf growth.

The best stem growth in comparison to that of the control, was noticed in the experimental lot of the C.M. followed by substrates R.S. and G.R.

The best leaf growth in comparison to that of the control was noted in G.R. followed by C.M. and R.S., respectively.

For the plants in C.M., the stem growth was significantly higher than that of the leaves. A similar situation was also observed in R.S., with the mention that SS’CB’ exhibited the highest growth and one of the highest overall growths of all the experimental treatments.

## 3. Discussion

As already proven in various studies, the growth and development of plants are affected by temperature, soil moisture, and soil depth. The temperature of the environment is crucial, as different plants require specific temperature ranges for optimal growth. Extreme temperatures can harm plant growth, making it necessary to provide an appropriate environment. Water is also crucial and the soil moisture level affects the uptake of nutrients and minerals by plants. Soil that is too dry can cause stunted growth, while overly wet soil can cause root rot. Soil depth also plays a critical role since it can limit or facilitate root growth. Deeper soil leads to better root growth, stronger and healthier plants, and a more stable environment. Therefore, it is essential to provide the right balance of temperature, soil moisture, and soil depth for optimal plant growth and development.

The subsequent sections comprise aspects and discussions concerning the Impact of abiotic factors on the growth of the studied plants.

### 3.1. Root Growth Rate

Healthy growth of the root system is perhaps the most important aspect in the morphological evolution of a plant. This growth can be significantly affected by changes in soil temperature [27,28,29,30]. Thermal fluctuations, depending on the stage of development and the duration of exposure, can significantly affect the healthy growth of the roots and implicitly the root–shoot relationship [30,31]. Researchers have found that by optimizing the root zone temperature, plant growers could increase their plant production [32].

Research results showed that although root growth begins at 13 °C, the rate at which it happens can be relatively low at soil temperatures below 22 °C. In their case, most growth occurred during the summer when soil temperatures were above 27 °C. They concluded that soil temperatures below 22 °C limit root growth, thereby limiting overall plant evolution [33].

Our results, however, partly oppose Bevington’s findings. Most plants developed their root system during periods when temperatures were between 12 and 18 °C, and this behavior can be mostly accredited to the place the experimental plants originate from, i.e., North America and the Subalpine regions of the Caucasus [34], preferring cooler environments.

Irrigation is highly recommended during the growing season, but most *Sedum* species will survive if not watered for a month. In general, *Sedums* are more likely to be affected by over-watering than under-watering [34].

Even though further studies are needed regarding this topic, researchers found that understanding a plant’s place of origin and mimicking its natural habitat did not necessarily guarantee their survival on green roofs. They recommend watering the plants during extreme drought conditions to help them stay alive and healthy [35]. Interestingly, for succulent plants such as *Sedum*, the frequency of watering is more important than the amount of water given [36]. This means that while these plants have adapted to store water efficiently, they still require regular watering to ensure their survival. Moreover, the frequency of water administration can create a cooler microclimate, thus creating a more suitable environment for the plants, both under and above-ground.

Because water consumption in smaller plants is reduced, they become more tolerant to long periods of drought [37]. When there is no irrigation system, it is important to choose plants that can tolerate drought. If plants receive less water when they are first planted, their roots tend to be thinner but more active, which makes them better at surviving drought later on. In contrast, plants that might receive excessive amounts of water early on had bigger roots, but are less able to tolerate drought. So, it is important to gradually reduce watering to help plants acclimate to drought conditions, rather than suddenly stopping watering altogether [8].

Drought stress is known to induce a myriad of physiological changes in plants, where the first noticeable effects are a reduction in leaf water content and the degradation of cell membranes [38,39,40].

However, some species of plants, such as certain types of sedums, have evolved to thrive in arid environments and can survive for extended periods on rainwater alone, without any additional irrigation [41].

In our study, the root growth may have been affected by varying moisture levels in the culture substrates. It was observed that the humidity of the substrate fluctuated unevenly throughout the years, with the highest levels occurring during summer and the lowest during autumn and winter. Humidity levels fluctuated similarly to those of the temperature, indicating a correlation between soil humidity, soil temperature, and, therefore, possibly plant growth.

Peaks of up to 70–80% humidity levels were recorded during the summer in green roofs and commercial growing media. Most of the lower temperatures were recorded in the lot consisting of sand, with a range of 0.36–9.21%.

Most studies claim that plant growth is strongly affected by the depth of the growth substrate [12,42,43,44], and in most cases, substrates with shallow depths can only be tolerated by plants such as succulents [45,46,47].

Studies show that a deeper substrate can retain more water, which allows plants to maintain a better physiological state when additional watering is restricted on green roofs [12,48]. Therefore, a deeper substrate tends to promote the growth rate, survival, and growth of *Sedum* species [12,45,49]. A deeper substrate can help maintain higher levels of both heat and humidity. This is because a thicker layer of the substrate has a greater capacity to hold water, which can help regulate the humidity within the habitat. Additionally, a deeper substrate can also act as an insulator, helping to maintain a more stable temperature within the habitat.

In our specific case, both G.R. and C.M. presented similar temperatures and humidity levels. Because of its compositional characteristic, sand presented lower humidity levels over the years but with a higher overall temperature average.

Following the analyses carried out for this paper and according to the presented data, we can consider that the plant growth was not negatively affected by pedoclimatic conditions such as substrate depth, temperature, or humidity.

Comparing the root growth of each plant between years, it appears that there was generally a decrease in root growth in the second year compared to the first year. Additionally, the monthly average difference in root growth between years was generally negative, indicating a decrease in root growth over time.

### 3.2. Stem Growth Rate

In our study, the stem growth in the case of the commercial mix compared to sand was 44% higher in *Sedum spurium* ‘Purpur Winter’, 51% higher in *Sedum spathulifolium* ‘Cape Blanco’ and 44% higher in *Sedum spathulifolium* ‘Purpureum’.

In comparison, for 12 months, studies were conducted using five of the most commonly used *Sedum* species for green roofs. The researchers concluded that plants exposed to an average annual temperature of 14.8 °C, rainfall of 258 mm, and soil moisture of 69% showed plant dimensions between 169 cm^2^ and 644 cm^2^ with an average of 307 cm^2^. This gave them an average monthly growth rate of 0.73 cm [50].

Other studies showed that, in 4 weeks, *Echeveria* plants averaged 41.80 mm in height and 79.74 mm in diameter, resulting in a growth rate, per day, of 1.49 mm and 2.84 mm, respectively [51].

### 3.3. Morphological Analysis

Plant tolerance to drought can be assessed by measuring physiological characteristics under water and nutrient stress. Results from a roof garden study [52] also indicated that total biomass reduction was important for the survival of the *Sedum* species studied under drought stress conditions.

Drought stress creates a wide range of physiological changes, starting with a decrease in leaf water content, thus becoming a good indicator of the overall hydration status of plants [39,40]. Cell membranes are prime targets of drought stress [38].

Studies in which researchers attempted to determine the growth behavior of plants under controlled drought conditions showed that after 90 days, in 10 cm deep substrates, *Sedum* shoots weighed an average of 1.62 g and roots weighed 0.18 g, giving a total of 1.80 g. In the first experiment of this study, it was observed that *Sedum lineare* plant size was smaller in the deeper substrate (10 cm) than in the shallower substrate (4 cm) [14].

Similarly, researchers conducted a study on *Sedum zokuriense* in which under controlled 50% shade, regular watering but no fertilization, with an average temperature and humidity of 25.8 ± 7.9 °C and 69.8 ± 16.7%, respectively, resulted in better plant growth—4.28 g shoot and 0.58 g fresh root mass. On average, plants measured 11.45 cm long and 7.90 cm wide. Additionally, 65% shading resulted in the tallest plants, while the highest shoot and root fresh weights were observed in plants grown in river sand, vermiculite, and perlite (6:2:2). The use of decomposed granite, fertilizer-amended media, and perlite (5:2:3) as a potting medium had the lowest survival rate, while those grown under other treatments integrated with compost or organic materials had 70–97% survival rates. In their study, fertilization rates did not statistically affect plant height, width, and fresh weight of shoot parameters. Overall, the study suggests that *Sedum zokuriense* can tolerate shaded conditions of up to 80%, and well-established and mature stonecrop plants can withstand extremely low temperatures and limited watering. The use of sandy soil coupled with compost has been reported to protect *Sedum* spp. From rot and facilitate proper drainage and aeration [53].

One study noted that the optimal water regime in which *Sedum* species retain all their specific morphological characteristics is three days between waterings. The growing substrate used was a sandy substrate with a saturation percentage of 73.79% and a pH of 7.6. *Sedum spurium* showed the highest survival percentage with 91.5% at a watering interval of six days [54]. When stressed, plants tend to adapt to their environment and the most affected parts are leaves, offshoots, and stems [55].

**Figure 9:**
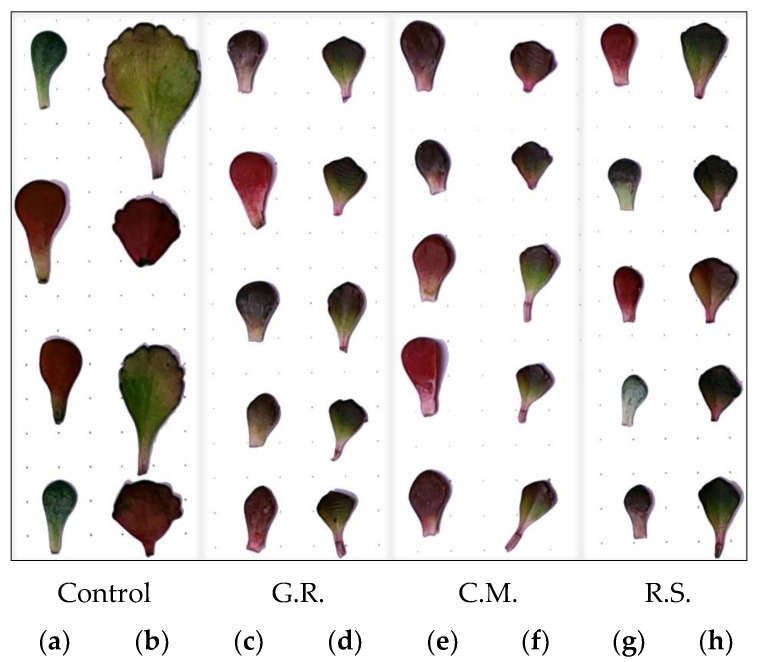
Overall leaf growth and comparison for *Sedum* plants under abiotic stress factors: control (**a**)—*Sedum spathulifolium*, (**b**)—*Sedum spurium*; green roof substrate (G.R.), (**c**)—*Sedum spathulifolium*, (**d**)—*Sedum spurium*; commercial mix (C.M.), (**e**)—*Sedum spathulifolium*, (**f**)—*Sedum spurium*; river sand (R.S.), (**g**)—*Sedum spathulifolium*, (**h**)—*Sedum spurium*.

As seen in Figure 9, in the case of leaves, their sizes vary much more strongly. Plants studied in the sand substrate (R.S.) presented 68% smaller values than the control, commercial mix (C.M.) 60%, and green roof (G.R.) 49%.

Sedums are known for their attractive, fleshy foliage, which typically ranges from green to red, and their delicate, star-shaped flowers that bloom in clusters. Drought and the lack of nutrients can affect the aesthetic value of sedums because they are succulent plants that store water in their leaves and stems. When sedums are subjected to drought, they can become dehydrated, causing their leaves to wilt and lose their plump, full appearance. In extreme cases, the leaves can even fall off the plant. Similarly, when sedums lack essential nutrients, their leaves may lose their specific color, which can significantly diminish their visual appeal.

In the case of our study, the chromatic characteristics were preserved but this aspect alone does not create the desired overall image in a landscape design. The leaves show strong leaf polymorphism and poor overall growth, which, in turn, creates a visually desolate appearance.

## 4. Materials and Methods

### 4.1. Experimental Site and Pedoclimatic Conditions

These studies were conducted in Cluj-Napoca, Romania, specifically on the south-facing terrace of the Institute of Advanced Horticultural Research of Transylvania, located on the campus of the University of Agricultural Sciences and Veterinary Medicine of Cluj-Napoca.

From a climactic point of view [56], Cluj-Napoca has a moderate continental climate with oceanic influences. Temperatures and precipitations can vary across the city’s regions due to different elevation ranges. The temperatures are influenced by solar radiation and dynamic and geographical factors.

In 2020 and 2021, during the studies, Cluj-Napoca had an average annual temperature of 10.6 °C and 9.8 °C, respectively, and received a total of 1114.47 mm of precipitation, divided almost equally between the years.

**Figure 10:**
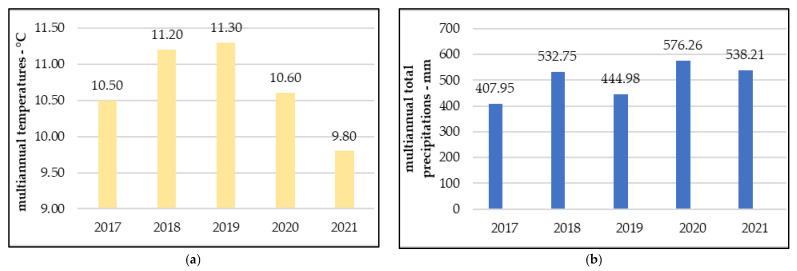
(**a**) Multiannual average temperatures; (**b**) multiannual total precipitations.

Although annual temperatures gradually increased by an average of 0.8 °C over the course of 2017, 2018, and 2019, this trend stopped in the following years and the annual averages decreased to 10.6 °C in 2020 and 9.8 °C in 2021, respectively (Figure 10a). Precipitation in Cluj-Napoca can be characterized as unevenly distributed, alternating from one year and region to another without presenting a stable pattern (Figure 10b).

In order to carry out the desired studies, some decisions had to be made in advance. The decisions included the choice of substrate and containers in which to place the plants, the type of structure from which the plants would be suspended, and the type of *Sedum.*

The three growth substrates used were as follows:

- specific substrate for green roofs, typical for use as a growing medium for semi-intensive green roofs (G.R.);
- a commercial mix of topsoil with traces of dolomite and perlite (C.M.);
- river sand.

To better understand how the different growth media could affect the studied plants, the following characteristics were analyzed, and the results are presented in Table 13.

- pH: method used—potentiometric;
- nitrogen (N): method used—Kjedahl;
- phosphorus (P): method used—colorimetric;
- potassium (K): method used—flamphotometric;
- particle size analysis: method used—Kacinscki;
- hygroscopicity coefficient: method used—Mittscherlich;
- carbonates: method used—Scheibler;
- electrical conductivity: method used—conductometric.

For the green roof substrate (G.R.), the pH was weakly alkaline with a very low supply of nitrogen, a good supply of phosphorus, and a very good supply of potassium. The carbonate content was low and the hygroscopicity coefficient and textures were typical for clay soils. In terms of electrical conductivity, the values indicated a very high content of soluble salts.

For the commercial mix substrate (C.M.), the pH was acidic with a very good supply of nitrogen, a very good supply of phosphorus, and a very good supply of potassium. The charcoal analysis did not give any results, and the hygroscopic coefficient was specific for a sandy soil with a specific clay texture. In terms of electrical conductivity, the values indicated a very high content of soluble salts.

For the river sand substrate (R.S.), the pH was weakly alkaline, lacking nitrogen, with a poor supply of phosphorus and a very good supply of potassium. The carbonate content was medium with a hygroscopicity coefficient and texture typical of sand. In terms of electrical conductivity, the values indicated a low content of soluble salts.

Analyzing the selected substrates helped us determine their texture, the presence of organic matter, structure, and water-holding capacity. Understanding the soil’s drainage characteristics is essential in preventing waterlogging or excessive drying, both of which can impact plant health. Soil humidity not only provides a water reserve for plants but can also reduce or increase the difference in soil temperature between day and night, thus protecting plant roots from extreme temperature variations. As the moisture content of the soil increases, the temperature level also increases, thus resulting in a higher heat storage capacity [57]. Soil temperature affects biological, physical, and chemical processes in the soil and thus the healthy growth of plants [58].

### 4.2. Plant Material and Design

Three different *Sedum* varieties were studied—*Sedum spurium* ‘Purpur Winter’ (SS’PW’), *Sedum spathulifolium* ‘Cape Blanco’ (SS’CB’), and *Sedum spathulifolium* ‘Purpureum’ (SS’P’). Both *Sedum* species, *Sedum spurium* and *Sedum spathulifolium,* are native to cool and chilly climates, usually found in North America and the Subalpine regions of the Caucasus [34].

In total, 12,276 cuttings, 2046 pots, and 3 m³ of growing substrate were needed to complete the entire arrangement:

- 482 pots, respectively, 2892 cuttings of *S. spurium* ‘Purpur Winter’;
- 983 pots, respectively, 5898 cuttings of *S. spathulifolium* ‘Cape Blanco’;
- 581 pots, respectively, 3486 cuttings of *S. spathulifolium* ‘Purpureum’.

Following the specialized literature on succulent propagation [3,15,59] in order to create a common starting point for the plants, stem cuttings were used.

Their specific metabolism makes succulents drought-resistant, for the most part, being able to grow in any type of substrate. In addition, they are species that prefer low-nutrient soils and grow very well in light, well-drained soils.

For plant preparation, 4–7 cm long cuttings were prepared and planted, 6 per 17 cm wide and 13 cm deep pot. In order to avoid damaging the plants, wooden tweezers were used.

The pots were divided equally and filled with three types of substrates: green roof substrate, a commercial mixture of topsoil with traces of dolomite and perlite, and river sand. The pots were then suspended on a specially made metal structure.

**Figure 11:**
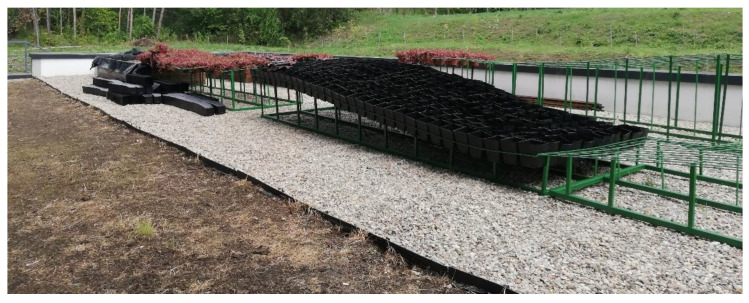
Welded mesh structures with differentiating heights.

The six metal structures were made according to an original concept, from welded mesh, each with dimensions of 110 × 400 cm in width and length and metal profiles for support, cut to different lengths to create the wavy effect of the “work tables”. As seen in Figure 11, the height of the structure varied from 16 to 70 cm.

### 4.3. Analytical Methodology and Data Processing

For the present study, emphasis was placed on establishing the rate at which *Sedum* roots and stems grow and their overall morphological characteristics after a fixed period. The three analytical components reprising the present study, result in a comprehensive analysis of the behavior of different *Sedum* varieties under different pedo-climatic conditions and abiotic stresses (water and nutrients).

For 20 months, specifically from May 2020 to December 2021, a variety of technical tools were utilized to conduct observations and measurements: digital caliper; metal ruler or roulette; measuring probe for soil temperature and humidity (IN/OUT Temp./RH SD Card Logger—87799).

**Figure 12:**
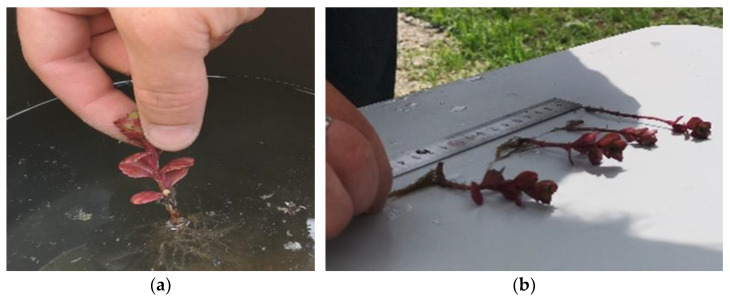
(**a**,**b**) Plant preparation for measuring their growth progress.

To collect the data and subsequently perform the analyses, at a 7-day interval, cuttings from each plant group were removed from their substrates, cleaned, washed of impurities with clean tap water, and placed on a work table (Figure 12a,b).

The plants were positioned to measure their morphological characteristics using a metal ruler or caliper. Additionally, during these 7-day intervals, with the help of a digital probe, the internal temperature and humidity of each substrate were measured.

To determine the rate at which our plants developed in comparison to their natural habitat, based on previous documented observations and research [34], a monthly expected growth rate (E.G.R.) of 1.33 cm was chosen for *Sedum spurium* and 0.53 cm for *Sedum spathulifolium*. At the end of the experiment, the E.G.R. was compared with the actual growth rate (A.G.R.). These values were expressed in percentages and related to the average soil temperatures and humidity.

In order to conduct the morphological measurements, 12 samples of 100 plants were evaluated—3 control and 9 experimental. This approach was essential to determine the impact of water and nutrient stress on plant growth. Similar to the experimental samples, one hundred stems of each variety were randomly collected for the control group, resulting in a total of three hundred stems. The numbers of leaves were as follows: 1800 leaves of S.S.’PW’—*Sedum spuirum* ‘Purpur Winter’; 1584 leaves of S.S.’CB’—*Sedum spathulifolium* ‘Cape Blanco’; 1760 leaves of S.S.’P’—*Sedum spathulifolium* ‘Purpureum’.

The control groups were planted at the same time as the studied plants in typical garden soil. Climatic conditions were identical to those of the experimental treatments.

The following morphological traits were measured:

- stem weight;
- stem length and diameter;
- number of leaves;
- leaf weight;
- leaf surface area.

In total, 1200 stems and 14,057 leaves were cataloged.

### 4.4. Statistical Analysis

In order to investigate whether there were any statistically significant differences between the experimental treatments, for this research paper, we performed the DUNCAN test and variance analysis with a confidence level of 95% to further consolidate our findings.

## 5. Conclusions

In conclusion, we could confirm that there were significant differences between the experimental treatments—green roof (G.R.), commercial mix (C.M.), river sand (R.S.)—and plant varieties—*Sedum spurium* ‘Purpur Winter’ (SS’PW’), *Sedum spathulifolium* ‘Cape Blanco’ (SS’CB’), and *Sedum spathulifolium* ‘Purpureum’ (SS’P’). The results of this study suggest that pedo-climatic conditions such as substrate type, depth, temperature, and humidity can, but did not necessarily, impact plant growth. There was an overall decrease in root growth between the years, resulting in the need for continued monitoring of plant growth over time. The stem growth in the commercial mix C.M. was higher than that of the plants in R.S. and green roof substrate G.R., as demonstrated by the increased percentage values.

All experimental treatments showed negative differences in their growth compared to those of the control with the overall worse results being in the river sand (R.S.) treatment.

Although the chromatic characteristics were preserved, the leaves’ strong polymorphism and the plants’ overall poor growth created a desolate appearance, which may not be desirable in future landscape designs. As many plants failed to survive, the need to evaluate plants under field conditions for green roof use in new climates beyond these approaches was highlighted.

In regions characterized by hot and arid climates with severe water scarcity and no irrigation, it is advisable to opt for plants, such as sedums, that possess specific hardiness traits for green roofs. When it comes to selecting plants, it is imperative to take into account not just their place of origin and theoretical attributes but also real-life examples and uses.

## Figures and Tables

**Table 1:** The effect of substrate humidity and temperature on the root growth rate in green roof substrates. Different letters indicate significant differences between experimental treatments (*p* < 0.05).

Specification		Length, cm			Substrate Humidity %	Substrate Temperature °C
		S.S.’PW’	S.S.’CB’	S.S.’P’		
2020	1st year total	7.60 ± 0.38	8.26 ± 0.41	6.68 ± 0.33	35.41	17.59
	monthly average	1.09 ± 0.05	1.18 ± 0.06	0.95 ± 0.05		
2021	2nd year total	7.17 ± 0.36	7.96 ± 0.40	8.63 ± 0.43	26.83	12.35
	monthly average	0.80 ± 0.04	0.88 ± 0.04	0.96 ± 0.05		
20-month average		0.92 ± 0.05 d	1.01 ± 0.05 b	0.96 ± 0.05 c	30.26	14.45
TOTAL		14.77 ± 0.74	16.22 ± 0.81	15.31 ± 0.77		

**Table 2:** The effect of substrate humidity and temperature on the root growth rate in a commercial soil mix. Different letters indicate significant differences between experimental treatments (*p* < 0.05).

Specification		Length, cm			Substrate Humidity %	Substrate Temperature °C
		S.S.’PW’	S.S.’CB’	S.S.’P’		
2020	1st year total	8.97 ± 0.45	6.92 ± 0.35	5.11 ± 0.26	38.91	16.63
	monthly average	1.28 ± 0.06	0.99 ± 0.05	0.73 ± 0.04		
2021	2nd year total	7.71 ± 0.39	7.06 ± 0.35	6.76 ± 0.34	31.29	12.63
	monthly average	0.86 ± 0.04	0.78 ± 0.04	0.75 ± 0.04		
20-month average		1.04 ± 0.05 a	0.87 ± 0.04 e	0.74 ± 0.04 g	34.34	14.23
TOTAL		16.68 ± 0.83	13.98 ± 0.70	11.87 ± 0.59		

**Table 3:** The effect of substrate humidity and temperature on the root growth rate in river sand. Different letters indicate significant differences between experimental treatments (*p* < 0.05).

Specification		Length, cm			Substrate Humidity %	Substrate Temperature °C
		S.S.’PW’	S.S.’CB’	S.S.’P’		
2020	1st year total	6.14 ± 0.31	7.09 ± 0.35	6.14 ± 0.31	16.07	18.52
	monthly average	0.88 ± 0.04	1.01 ± 0.05	0.88 ± 0.04		
2021	2nd year total	2.57 ± 0.13	7.12 ± 0.36	6.06 ± 0.30	11.00	13.56
	monthly average	0.29 ± 0.01	0.79 ± 0.04	0.67 ± 0.03		
20-month average		0.54 ± 0.03 h	0.89 ± 0.04 e	0.76 ± 0.04 f	13.03	15.55
TOTAL		8.71 ± 0.44	14.21 ± 0.71	12.20 ± 0.61		

**Table 4:** The effect of substrate humidity and temperature on the stem growth rate in green roof substrates. Different letters indicate significant differences between experimental treatments (*p* < 0.05).

Specification		Length, cm			Substrate Humidity %	Substrate Temperature °C
		S.S.’PW’	S.S.’CB’	S.S.’P’		
2020	1st year total	4.30 ± 0.22	1.65 ± 0.08	1.25 ± 0.06	35.41	17.59
	monthly average	0.61 ± 0.03	0.24 ± 0.01	0.18 ± 0.01		
2021	2nd year total	1.52 ± 0.08	1.27 ± 0.06	1.21 ± 0.06	26.83	12.35
	monthly average	0.19 ± 0.01	0.16 ± 0.01	0.15 ± 0.01		
20-month average		0.39 ± 0.02 c	0.19 ± 0.01 ef	0.16 ± 0.01 f	30.26	14.45
TOTAL		5.82 ± 0.29	2.92 ± 0.15	2.46 ± 0.12		

**Table 5:** The effect of substrate humidity and temperature on the stem growth rate in commercial mix soil. Different letters indicate significant differences between experimental treatments (*p* < 0.05).

Specification		Length, cm			Substrate Humidity %	Substrate Temperature °C
		S.S.’PW’	S.S.’CB’	S.S.’P’		
2020	1st year total	6.10 ± 0.31	2.63 ± 0.13	2.74 ± 0.14	38.91	16.63
	monthly average	0.87 ± 0.04	0.38 ± 0.02	0.39 ± 0.02		
2021	2nd year total	6.90 ± 0.35	3.33 ± 0.17	2.50 ± 0.13	31.29	12.63
	monthly average	0.86 ± 0.04	0.42 ± 0.02	0.31 ± 0.02		
20-month average		0.87 ± 0.04 a	0.40 ± 0.02 c	0.35 ± 0.02 d	34.34	14.23
TOTAL		13.00 ± 0.65	5.96 ± 0.30	5.24 ± 0.26		

**Table 6:** The effect of substrate humidity and temperature on the stem growth rate in river sand. Different letters indicate significant differences between experimental treatments (*p* < 0.05).

Specification		Length, cm			Substrate Humidity %	Substrate Temperature °C
		S.S.’PW’	S.S.’CB’	S.S.’P’		
2020	1st year total	4.64 ± 0.23	1.52 ± 0.08	1.54 ± 0.08	16.07	18.52
	monthly average	0.66 ± 0.03	0.22 ± 0.01	0.22 ± 0.01		
2021	2nd year total	2.70 ± 0.14	1.41 ± 0.07	1.38 ± 0.07	11.00	13.56
	monthly average	0.34 ± 0.02	0.18 ± 0.01	0.17 ± 0.01		
20-month average		0.49 ± 0.02 b	0.20 ± 0.01 ef	0.19 ± 0.01 ef	13.03	15.55
TOTAL		7.34 ± 0.37	2.93 ± 0.15	2.92 ± 0.15		

**Table 7:** Morphological plant analysis for *Sedum* varieties after 20 months of hydric and nutritive stress. Arrow orientations indicate whether the values are lower or higher than those of the control. Different letters indicate significant differences between experimental treatments (*p* < 0.05).

	Control			Green Roof Substrate						Commercial Mixture						River Sand					
	SS’PW’	SS’CB’	SS’P’	SS’PW’		SS’CB’		SS’P’		SS’PW’		SS’CB’		SS’P’		SS’PW’		SS’CB’		SS’P’	
Σ leaves	1800	1584	1760	905		1602		1515		1051		1528		511		511		706		584	
Σ stems	100	100	100	100		100		100		100		100		100		100		100		100	
stem weight g	0.337 ± 0.017 A	0.169 ± 0.008 D	0.183 ± 0.009 C	0.100 ± 0.005 F		0.068 ± 0.003 I		0.071 ± 0.004 I		0.192 ± 0.010 B		0.083 ± 0.004 H		0.092 ± 0.005 G		0.106 ± 0.005 E		0.054 ± 0.003 J		0.048 ± 0.002 J	
	100%	100%	100%	70%	↓	60%	↓	61%	↓	43%	↓	51%	↓	50%	↓	68%	↓	68%	↓	74%	↓
leaf weight g	0.030 ± 0.002 B	0.062 ± 0.003 A	0.064 ± 0.003 A	0.021 ± 0.001 C		0.032 ± 0.002 B		0.032 ± 0.002 B		0.017 ± 0.001 C		0.024 ± 0.001 C		0.025 ± 0.001 C		0.017 ± 0.001 C		0.024 ± 0.001 C		0.024 ±0.001 C	
	100%	100%	100%	32%	↓	49%	↓	50%	↓	45%	↓	61%	↓	61%	↓	45%	↓	61%	↓	62%	↓
stem length cm	15.032 ± 0.752 A	5.260 ± 0.263 C	5.852 ± 0.293 B	4.100 ± 0.205 D		1.595 ± 0.080 F		1.376 ± 0.069 F		5.954 ± 0.298 B		2.077 ± 0.104 E		2.005 ± 0.100 E		5.050 ± 0.253 C		2.003 ± 0.100 E		1.980 ± 0.099 E	
	100%	100%	100%	73%	↓	70%	↓	76%	↓	60%	↓	61%	↓	66%	↓	66%	↓	62%	↓	66%	↓
stem diam. cm	0.236 ± 0.012 C	0.239 ± 0.012 B	0.240 ± 0.012 B	0.190 ± 0.010 G		0.237 ± 0.012 B		0.186 ± 0.009 G		0.223 ± 0.011 E		0.227 ± 0.011 D		0.192 ± 0.010 G		0.216 ± 0.011 E		0.265 ± 0.013 A		0.211 ± 0.011 F	
	100%	100%	100%	19%	↓	1%	↓	22%	↓	5%	↓	5%	↓	20%	↓	8%	↓	11%	↑	12%	↓
leaf surface cm^2^	1.436 ± 0.072 A	0.790 ± 0.040 C	0.843 ± 0.042 B	0.345 ± 0.017 F		0.442 ± 0.022 E		0.505 ± 0.025 D		0.361 ± 0.018 F		0.375 ± 0.019 F		0.302 ± 0.015 G		0.211 ± 0.011 H		0.209 ± 0.010 H		0.178 ± 0.009 H	
	100%	100%	100%	76%	↓	44%	↓	40%	↓	75%	↓	52%	↓	64%	↓	85%	↓	74%	↓	79%	↓

**Table 8:** Variance analysis for stem weight. Different letters indicate significant differences between experimental treatments; 000 indicates very significant negative values at *p* < 0.05.

Specification		Weight, Grams	%	Difference	Significance
SS’PW’	control	0.34 A	100	0	
	G.R.	0.10 F	29.50	−0.24	000
	C.M.	0.19 B	56.80	−0.15	000
	R.S.	0.11 E	31.50	−0.23	000
SS’CB’	control	0.17 D	100	0	
	G.R.	0.07 I	40.10	−0.10	000
	C.M.	0.08 H	49.10	−0.09	000
	R.S.	0.05 J	31.90	−0.12	000
SS’P’	control	0.18 C	100	0	
	G.R.	0.07 I	39.00	−0.11	000
	C.M.	0.09 G	50.30	−0.09	000
	R.S.	0.05 J	26.10	−0.14	000

**Table 9:** Variance analysis for leaf weight. Different letters indicate significant differences between experimental treatments; 000 indicates very significant negative values at *p* < 0.05.

Specification		Weight, Grams	%	Difference	Significance
SS’PW’	control	0.03 B	100	0	
	G.R.	0.02 C	68.20	−0.01	000
	C.M.	0.02 C	54.60	−0.01	000
	R.S.	0.02 C	54.60	−0.01	000
SS’CB’	control	0.06 A	100	0	
	G.R.	0.03 B	50.07	−0.03	000
	C.M.	0.02 C	39.10	−0.04	000
	R.S.	0.02 C	38.60	−0.04	000
SS’P’	control	0.06 A	100	0	
	G.R.	0.03 B	49.70	−0.03	000
	C.M.	0.02 C	38.70	−0.04	000
	R.S.	0.02 C	38.20	−0.04	000

**Table 10:** Variance analysis for stem length. Different letters indicate significant differences between experimental treatments; 000 indicates very significant negative values at *p* < 0.05.

Specification		Length, cm	%	Difference	Significance
SS’PW’	control	15.03 A	100	0	
	G.R.	4.10 D	27.30	−10.93	000
	C.M.	5.95 B	39.60	−0.15	000
	R.S.	5.12 C	34.00	−0.23	000
SS’CB’	control	5.26 C	100	0	
	G.R.	1.59 F	30.30	−0.10	000
	C.M.	2.08 E	39.50	−0.09	000
	R.S.	2.00 E	38.10	−0.12	000
SS’P’	control	5.85 B	100	0	
	G.R.	1.38 F	23.50	−0.11	000
	C.M.	2.01 E	34.30	−0.09	000
	R.S.	1.98 E	33.80	−0.14	000

**Table 11:** Variance analysis for stem diameter. Different letters indicate significant differences between experimental treatments; 0, 000, *** indicate significant and very significant negative and positive values at *p* < 0.05.

Specification		Diameter, cm	%	Difference	Significance
SS’PW’	control	0.24 C	100	0	
	G.R.	0.19 G	80.60	−0.24	000
	C.M.	0.22 E	94.50	−0.15	000
	R.S.	0.22 E	91.70	−0.23	000
SS’CB’	control	0.24 B	100	0	
	G.R.	0.24 B	99.00	−0.10	0
	C.M.	0.23 D	94.80	−0.09	000
	R.S.	0.27 A	110.90	−0.12	***
SS’P’	control	0.24 B	100	0	
	G.R.	0.19 G	77.60	−0.11	000
	C.M.	0.19 G	79.90	−0.09	000
	R.S.	0.21 F	87.90	−0.14	000

**Table 12:** Variance analysis for leaf surface. Different letters indicate significant differences between experimental treatments; 000 indicates very significant negative values at *p* < 0.05.

Specification		Surface, cm^2^	%	Difference	Significance
SS’PW’	control	1.44 A	100	0	
	G.R.	0.35 F	24.00	−0.24	000
	C.M.	0.36 F	25.20	−0.15	000
	R.S.	0.21 H	14.70	−0.23	000
SS’CB’	control	0.79 C	100	0	
	G.R.	0.44 E	55.90	−0.10	000
	C.M.	0.38 F	47.50	−0.09	000
	R.S.	0.21 H	26.50	−0.12	000
SS’P’	control	0.84 B	100	0	
	G.R.	0.51 D	60.00	−0.11	000
	C.M.	0.30 G	35.80	−0.09	000
	R.S.	0.18 H	21.10	−0.14	000

**Table 13:** Chemical compound (macro elements) and structural analysis of the substrates used in the study.

Growth Media Analysis													
Crt. No.	Substrate	pH	N	P	K	Hy	CaCO₃	El. Cond.	Particle Size Analysis				
			%	ppm	ppm		%	mS	Coarse Sand	Fine Sand	Dust I	Dust II	Clay
1	G.R.	8.01	0.038	69	810	3.22	1.7	2.27	8.67	64.5	5.18	8.89	12.76
2	C.M.	4.92	1.31	3800	4740	1.75	-	3.76	1.25	58.1	3.3	10.85	26.5
3	R.S.	8.82	0	7	254	0.41	4.1	0.31	8.86	83.96	1.3	0.57	5.31

## Data Availability

Not applicable.
